# From the 20th of August to the 20th of September 1799; Being the Result of the Public and Private Practice of a Physician at the West End of the Town

**Published:** 1799-12

**Authors:** 


					408 Lift of Difeafes in London,
LIST OF DISEASES IN LONDON,
From the 20th of Augujl to the 20th of September 1799; being the refult of
the public and private Prailice of a Phyjician at the W<:Ji End of the
9To<wn,
No. of Cafes.
ACUTE DISEASES.
Contagious malignant Fever 19
Meafles - - - 14
Scarlatina 2
Small-pox 4
.Hooping Cough 3
Catarrh - - io
Pneumonic Inflammation - 4
Acute Rheumatifm - 3
Eryfipelas - I .
Epistaxis 1
Ha:moptoe - - 2
Hsematemefis - - 1
Inteftinal Hemorrhagy - 1
Heftic - - 4
Synochus, or Summer Fever 12
Cholera - 2
Slow Fever 5
Acute Difeafes of Infants 15
Childbed and Milk Fevers 3
Aphthous Fever 2
Hydrophobia 1
CHRONIC DISEASES.
Cough and Difpncea - 26
Phthiiis pulmonalis - 10
Pleurodyne - 1
No. of Cafes.
Chronic Rhcumatifm - 5
Afthenia 19
Anafarca ... 8
Paralyfia 2
Dyfpepfia 20
Gaftrodynia - - - 11
Enterodynia, and Colic - 10
Conftipatio - - - 3
Bilious Vomiting, and Di-
arrhoea - - 22
Chlorofis and Amenorrhea 15
Fluor Albus - 4
Menorrhagia - 3
Prolapfus Uteri 1
Scirrhus of the Uterus - 2
Scirrhus of the Liver - 1
Jaundice 2
Tabes Mefenterica - - 3
Worms 3
Dyfury and Gravel - - 5
Lepra - - 3
Shingles 2
Nettle Ra(h - - - I
Erythema 1
Itch - 6
Porrigo - 3
The number of contagious fevers lias been much increafed during the
month of September, either from a continuance of the caufes affigned in.
the Journal, No. VIII. or from the general effect of the autumnal feafon
on the human body, by which it is rendered more than ufually fufccptible
of almoft every fpecies of infcflion. This cfFcvSt is exemplilied in the
above ila'temcnt of difeafes, and further proved by the obfervation of
practitioners
State of Difeafes in London.
409
practitioners differently flationed ; all of whom agree, that, along with
malignant fevers, the meafles, fmall-pox, fcarlatina, &c. have begun to
fpre.vd rapidly and widely.
Perfons expofed, without fhelter, to the vicifTitudes of the atmofphere,
have been affefted with the fynochus, with eryfipelas, pneumonic inflam-
mation, diarrhoea, cholera, and violent pain or inflammation of the
bowels, diforders often endangering life in the prefent feafon of the
year.
The cafe of hydrophobia occurred about the middle of Augufl; the
patient, a fine boy, ten years old, had been bitten by a dog in Fetter
Lane, fix weeks before the dread of water commenced. As he lived no
more than two days after the appearance of this fymptom, I had only an
opportunity of paying him a fingle vifit. His pulfe was then hurried
and irregular; his manner confufed and agitated; his utterance rapid
and abrupt; his eyes appeared bright and fparklirg, and had a mixed
expreffion of wildnefs and anxiety. He was perpetually hawking up
fome frothy phlegm, which feemed to irritate the larynx. When a glafs
of water was prefented to him, a rattling and convulfive motion took
place in his throat, rendering deglutition imprafticable: the water, whe-
ther applied to his lips, or merely put in his fight, feemed to excite eve-
ry mark of confternation and horror. All the above fymptoms could,
however, be produced by other means as ftrongly as by the application
of liquids. When Mr. Heather, the attending furgeon, attempted,
without any objection made by the patient, to examine the ftate of the
tonfils, &c. the fpoon no fooner approached his teeth, than the mufcles
of the throat were thrown into violent attion, and he made a noife,
"which was aptly enough compared by thofe around him, to the fnarling
of a fierce dog about to receive chaftifement. At the time of oar vifit to
this wretched boy, he was more compofed than he had been through ths
preceding night. He had, we were informed, had repeated fits of rav-
ing, in which he became almoft unmanageable, and endeavoured to bite
the hands of thofe who held him. In the evening after we faw him, he
began to complain of pain in the head, and of violent pains in the
ftomach and bowels: his fever and other fymptoms appeared to increafe;
and after enduring dreadful agony for feveral hours, he expired about two
o'clock in the morning. The body was examined by an attentive and ae-
rate obferver, Mr! Whatelv, of Bedford Row,.to whom the Editors
of the Medical and Phyftcal Journal are obliged for the following detail
of the appearances after death".
Number X.
F f f
On
> On opening the abdomen, all its contents appeared, to be in a found
ftate, except' the fpleen, which adhered to all the parts with which it
lies in contadt ; and was fmaller, and more convex, on its external fide,
than it is ufually found to be. Thefe appearances in the fpleen, had
been evidently produced by an inflammation of that organ, fome time
before the illnefs, which was the immediate caufe of the patient's death.
From the external appearance of the ftomach, it would have been thought
free from difeafe; but on opening it, the whole of its villous coat was
found to be greatly inflamed. The greateft degree of this inflammation
tvas at the large extremity of the Itomach, and particularly about the
fcardia, arourid which, to the extent of two or three inches, the villous
coat was abraded. The inflammation did not extend to any part of the
inteflinal canal : it ceafed at the pylorus; but it was continued from the
cardia, along the cefophagus, to the pharynx. About two inches of the
internal coat of that part of the cefophagus, which joins to the cardia,
Was alfo abraded. The inflammation upon the cefophagus, was not con-
fined merely to its internal furface, but reached to its external coat, on
which it was likewife continued through its whole extent; and in dif-
ferent parts of the ccllular membrane adjoining to it, finall quantities of
efFufed blood were found. The pharynx was very fligh'ly inflamed.
The uvula, palatum molle, and tonfils, were intirely free from inflam-
mation. The left lung was of a more folid texture, of a darker colour,
and fuller of blqod, than it is ever found to be in its natural ftate; all
which circumftances may be ccnfidered as the effe&s of inflammation.
About three or four ounces of bloody water were found in the right,
cavity of the cheft. There was likewife a flight inflammation on the
internal membrane of the trachea; but it ceafed near the larynx, which,
with the epiglottis, was intirely free from inflammation. There was
fome inflammation on the external furface of the heart, but it did not
extend to the inner furface of the ventricles.
Sept. 23, 1799.
R. W.

				

